# Slowly Growing Pulmonary Glandular Papilloma with Air Bronchogram: A Case Report

**DOI:** 10.5334/jbsr.3461

**Published:** 2024-02-19

**Authors:** Taehoon Lim, Jongsoo Park, Heejung Kwon

**Affiliations:** 1 Department of Pathology, Yeungnam University Medical Center, College of Medicine, Yeungnam University and Respiratory Center, 170 Hyeonchung-ro, Namgu, Daegu 42415, Republic of Korea; 2 Department of Radiology, Yeungnam University Medical Center, College of Medicine, Yeungnam University, Daegu, Korea; 3 Department of Pathology, Yeungnam University Medical Center, College of Medicine, Yeungnam University, Daegu, Korea

**Keywords:** Papilloma, solitary pulmonary nodule, lung neoplasms, growth, radiology, case report

## Abstract

Pulmonary glandular papilloma is a rare benign neoplasm that has not been studied extensively. This neoplasm presents as a solid nodule, consolidation, or mass, with or without atelectasis, and assessing the correlation between these findings and the risk of malignancy is challenging. A 60-year-old woman presented a solitary pulmonary nodule on screening chest radiography and chest computed tomography (CT). During the subsequent 2-year follow-up, CT showed a progressive increase in nodule size and an air bronchogram, suggesting malignancy. The patient underwent a right upper lobectomy, and the final diagnosis was glandular papilloma.

*Teaching point:* Pulmonary glandular papilloma with growth and an air bronchogram.

## Introduction

Lung glandular papilloma is a rare benign neoplasm. Solitary pulmonary papillomas are histologically classified as squamous, glandular, or mixed; the most common type is the squamous cell papilloma, whereas the glandular papilloma is the rarest [1,2]. On diagnostic images, papillomas present as solid nodules, consolidations, masses, or atelectasis [1–4]. Analyzing the association between these findings and the risk of malignancy is challenging due to the scarcity of data. This study presents the case of a slowly growing lung glandular papilloma with air bronchograms, suggesting malignancy.

## Case Report

**Figure 1:**
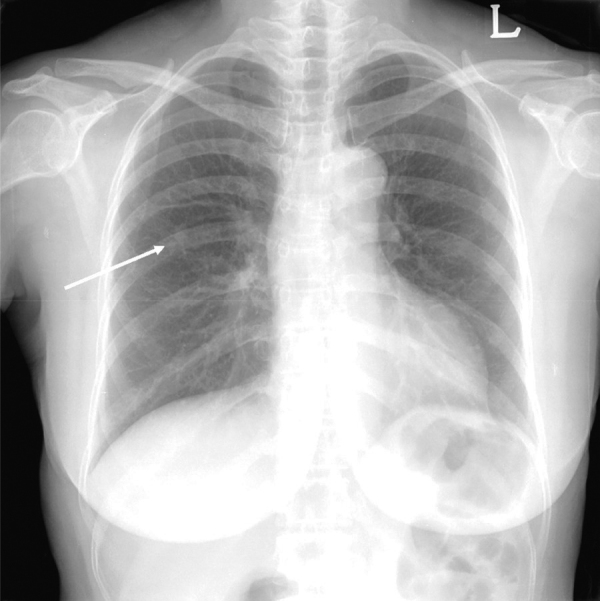
Initial chest radiograph shows an incidental nodule in the right upper lung field (arrow).

**Figure 2:**
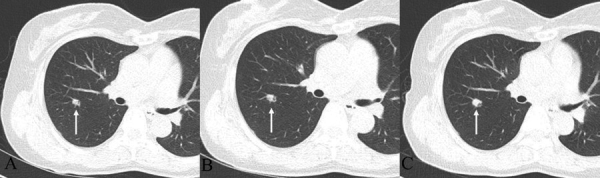
**Chest CT with follow up**. (A) 9-mm lobulated nodule with an internal air-bronchogram in the right upper lobe. (B) After 1 year, the nodule shows a minimal increase in size, 9 to 10 mm. (C) After 2 years later from the initial chest CT, the nodule changes from 10 to 13 mm in size, and the somewhat dilated internal bronchus are detected.

**Figure 3:**
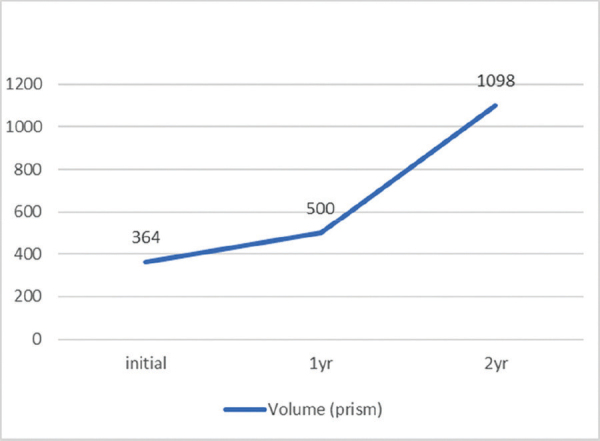
**Volume:** After 1 year, the volume doubling time from the initial state was 844.28 days, and the overall average volume doubling time after 2 years was 369.45 days.

A 60-year-old woman presented to the pulmonologist with a 9-mm solitary lobulated nodule in the right upper lobe as an incidental finding on screening chest radiography (Figure 1) and chest computed tomography (CT) (Figure 2A). All laboratory results were within normal limits. Follow-up CTs showed an increase in nodule size, from 9 mm initially to 10 mm after a year and 13 mm at the 2-year follow-up. The total volume doubling time (VDT) from the initial to the 2-year follow-up was 369.45 days, indicating a potential malignancy with a value below 400 days (Figure 3) [5]. Furthermore, air bronchograms became more evident and dilated, a feature known to be worrisome (Figure 2B, C). This growth and development of the air bronchogram suggest lung malignancy.

**Figure 4:**
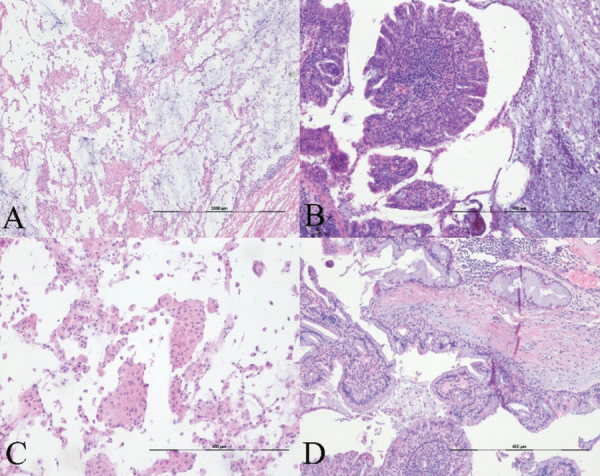
**Histopathology.** (A–B) Hematoxylin-eosin staining slide images show that the papillary tumor is constituted of pseudostratified columnar epithelium covering the fibrotic core. Surrounding alveolar spaces are filled with mucus on frozen (A, ×40) and permanent sections (B, ×100). (B) The lesion shows multiple papillary fronds lined by stratified and pseudostratified columnar cells. Moderate stromal lymphocytic infiltration and psammoma bodies are noted in the focal peripheral area. (C) In partial areas, small papillae with oncocytic change within the adjacent alveolar spaces are mimicking to the histologic features of the spread through the air spaces of pulmonary adenocarcinoma (×100). (D) Most of the lesion is composed of ciliated or non-ciliated columnar, goblet, and cuboidal cells. There are neither architectural atypia nor cytological atypia (×100).

Due to the vascular abutment and peripheral location of the tumor, an image-guided needle biopsy and bronchoscopy were not performed. Additionally, the close proximity to the pulmonary artery and the anticipated risk of significant hemorrhage precluded the possibility of conducting a needle biopsy. Therefore, the right upper lobectomy was performed via video-assisted thoracoscopic surgery. Frozen sections of the lesion showed a well-demarcated mass containing mucus-secreting oncocytes, cuboidal, and columnar cells (Figure 4A–D). The cells in the periphery of the tumor contained mucin and had lepidic growth. No cytologic atypia, mitosis, or necrosis were observed. Moderate stromal inflammation was present. Based on histological findings, the mass was confirmed to be glandular papilloma.

## Discussion

Pulmonary glandular papilloma is a rare benign neoplasm that affects the central tracheobronchial tree. It typically presents as a solitary nodule, mass, or consolidation, with or without atelectasis [1,3]. Glandular papillomas usually arise from the mucosal surface and appear as endobronchial lesions with luminal narrowing [1,6]. Radiologically, these neoplasms appear as solid nodules, as in the present case. Most of these lesions are benign and stable in size, indicative of benignity; however, some studies reported glandular papillomas that progressively enlarged, even rapidly [7]. Therefore, the diagnosis may be challenging.

In the assessment of solitary pulmonary nodules, some morphological characteristics, in addition to growth, indicate a high risk of malignancy, including spiculations, lobulations, air bronchograms, bubble-like radiolucency, and cystic airspaces [7]. Although this finding may be observed in benign and malignant cases, a dilated bronchus usually indicates malignancy, as in this case [7]. Therefore, the characteristics of this nodule suggest a high risk of malignancy due to the dilated air bronchogram and lobulated morphology.

Although a case presenting rapid growth was reported, the behavior of these tumors should be further studied. Specific procedures can improve diagnostic accuracy, including bronchoscopy, endobronchial ultrasound-guided transbronchial needle aspiration, and image-guided needle biopsy; nonetheless, in some cases, they were insufficient [3].

The glandular papilloma is composed uniform cytologically bland columnar cells admixed with occasional mucous cells that line inflamed, vascular, or hyalinized stromal core. The cytoplasm is usually clear. However, necrosis or mitosis is absent [8]. Consequently, surgical resection is often still needed to diagnose and treat a pulmonary glandular papilloma.

## Conclusion

Pulmonary glandular papilloma could be considered in patients with a solitary pulmonary nodule, which shows growth and an air-bronchogram.
